# Validity based on the response processes of the MMBGR Protocol Infants and Preschoolers: Instructional and Orofacial Myofuncional Clinical History

**DOI:** 10.1590/2317-1782/20242023109en

**Published:** 2024-05-31

**Authors:** Alexia Stéfanny Menezes da Conceição dos Santos, Yasmim Dourado Goes, Herick Santos Assis, Maria Vanessa Martins Alves, Amanda Tafnes Soares de Melo, Geovania Dias Barbora, Íkaro Daniel de Carvalho Barreto, Elisdete Maria Santos de Jesus, Giédre Berretin-Felix, Andréa Monteiro Correia Medeiros

**Affiliations:** 1 Departamento de Fonoaudiologia, Universidade Federal de Sergipe – UFS - São Cristóvão (SE), Brasil.; 2 Departamento de Fonoaudiologia, Faculdade de Odontologia de Bauru – FOB, Universidade de São Paulo – USP - Bauru (SP), Brasil.; 3 Prefeitura Municipal de São Cristóvão - São Cristóvão (SE), Brasil; 4 Centro Brasileiro de Pesquisa em Avaliação e Seleção e de Promoção de Eventos – CEBRASPE - Brasília (DF), Brasil.; 5 Faculdade de Ciências Farmacêuticas – FCF, Universidade Estadual de Campinas – UNICAMP - Campinas (SP), Brasil.; 6 Programa de Pós-graduação em Ciências da Saúde – PPGCS, Universidade Federal de Sergipe – UFS - São Cristóvão (SE), Brasil.

**Keywords:** Speech, Language and Hearing Sciences, Infant, Child, Preschool, Validation Study, Myofunctional Therapy, Stomatognathic System, Medical History Taking

## Abstract

**Purpose:**

Present the step of evidence of validity based on the responses to procedures of the MMBGR Protocol Infants and Preschoolers: Instructional and Orofacial Myofunctional Clinical History.

**Methods:**

Study developed according to phonoaudiologic tests validations recommendations. Validity analysis performed based on the process of instrument response. Ten speech therapists, that work on phonoaudiology clinic and/or orofacial myofunctional research on the population with age between 6 to 71 months, participated and applied the MMBGR Protocol Infants and Preschoolers: Instructional and Orofacial Myofunctional Clinical History with those responsible for the children. The speech therapists appraised the instrument applicability via Google®️ electronic forms, containing dichotic and/or multiple-choice questions, and likert scale with space to justify negative answers. The data was tabulated on Microsoft Excel 2016®️ worksheets and analyzed by the content validity index (CVI). The software R Core Team 2022 (Versão 4.2.2) was used.

**Results:**

All items from the MMBGR Protocol Infants and Preschoolers: Instructional and Orofacial Myofunctional Clinical History were valid when applied to real contexts. Orofacial Myofunctional Clinic history protocol- IVC 100% in terms of ease of application and filling and usage in professional practice; IVC 90% in terms of usefulness for phonoaudiology clinic. The instructional got IVC 80% in terms of clinic usefulness and 70% regarding to the prior reading necessity to fill the MMBGR Protocol Infants and Preschoolers.

**Conclusion:**

The Instrucional and Orofacial Myofunctional Clinical History, in the MMBGR Protocol Infants and Preschoolers had its validity proven based on the processes of responses to the usage on phonoaudiology clinic.

## INTRODUCTION

The collection of the patient's clinical history through the initial interview is of utmost relevance to the clinical practice of speech therapy, to obtain data regarding the complaint, developmental aspects, and biopsychosocial factors that may influence the individual's current health status^(1)^. This information guides the conduct, along with the clinical examination, according to the diagnosis established by the speech therapist^(1-4)^.

Currently, the use of standardized instruments in Orofacial Motricity (OM) clinics has enabled a quantifiable practice, capable of promoting increasingly reliable results^(5)^. For the age range between 6 months and 5 years and 11 months old, the MMBGR Protocol - Infants and Preschoolers has been proposed^(2,6)^.

The MMBGR Protocol - Infants and Preschoolers is composed of the Orofacial Myofunctional Clinical Examination (validated for test content, applicability, and reliability)^(6)^ and the Orofacial Myofunctional Clinical History and Instruction Protocol (validated for content and appearance^(2)^, though validation based on response processes has not yet been conducted).

Based on the above, the objective of this study is to present the stage of evidence of validity based on response processes^(7)^ for the MMBGR Protocol - Infants and Preschoolers: Orofacial Myofunctional Clinical History and Instruction^(2)^.

## METHODS

The descriptive study was approved by the Ethics Committee for Research Involving Human Subjects of the Federal University of Sergipe, under protocol number CAAE 12529419.6.0000.5546, opinion: 5,249,919. All participants (Speech Therapy professionals) and the guardians of infants and/or preschoolers signed the respective Informed Consent Forms (ICFs).

This is an analysis of validity based on response processes for the MMBGR Protocol - Infants and Preschoolers: Orofacial Myofunctional Clinical History and Instruction^(2)^, following validation study guidelines^(7)^. The applicability procedure was conducted by speech therapists (target population)^(7)^ using the protocol, along with guardians of children under 6 years old.

A total of 10 speech therapists from various regions of Brazil participated in the study. They were contacted via messaging apps and email after reviewing their curriculum vitae (*Lattes*) to verify their professional background in Speech Therapy and their experience in Orofacial Motricity (OM). Each name was preliminarily checked for visibility on social media and/or recognition for their work in OM. The inclusion criteria were being a speech therapist with experience, even if not exclusively, in OM and working with children under 6 years old. Exclusion criteria included participation in previous stages of the validation process for the MMBGR Protocol - Infants and Preschoolers, refusal to collaborate with the study, or failure to submit required documents on time.

Upon agreeing to participate in the research, each speech therapist received a link to an electronic form (Characterization - Part 1) regarding their personal background and professional trajectory (gender, age group, academic background, experience in OM, work with infants and/or preschoolers in OM, teaching experience, and their region of activity in Brazil). They were also asked about their use of protocols in clinical and research settings. The Instruction and Orofacial Myofunctional Clinical History Protocol^(2)^ belonging to the MMBGR Protocol - Infants and Preschoolers were provided via email in PDF format.

All participating speech therapists were instructed to use the instrument during their routine appointments, whether in speech therapy offices or clinics, in both public and private sectors. Each professional was to administer the instrument to a total of one to four caregivers of infants and/or preschoolers, who consented to participate by signing an Informed Consent Form (ICF).

After conducting the Anamnesis/Initial Interview with the patient's caregiver using the MMBGR Protocol - Infants and Preschoolers: Instruction and Orofacial Myofunctional Clinical History, each speech therapist provided their assessment through an electronic form (Evaluation of the application of the MMBGR Protocol - Infants and Preschoolers Clinical History - Part 2), using the link provided by the researchers. The speech therapists gave their opinions as planned in the validity based on response processes stage^(7)^, considering, for the Orofacial Myofunctional Clinical History Protocol: ease of application and completion, use in professional practice, usefulness for speech therapy clinics, and time required for application. For the Instructional part, they evaluated its usefulness in guiding the protocol completion and the need for prior reading before using the instrument.

The Likert scale responses corresponding to “strongly agree” and “agree partially” were considered agreement, with space for justification. The option “neither agree nor disagree” represented neutrality regarding the question. Conversely, responses corresponding to “disagree partially” and “strongly disagree” were considered disagreements.

The data collected via Google® electronic forms (parts 1 and 2) were tabulated in Microsoft Excel 2016® spreadsheets. Descriptive statistics were used for analysis, and the results were presented in percentages. Categorical variables were described using absolute frequency and relative percentage. The Content Validity Index (CVI) was calculated^(8)^. The hypothesis of non-inferiority of the CVI compared to a 70% agreement was tested using the exact binomial test (Wörz and Bernhardt, 2020)^(9)^. A significance level of 5% was adopted, and the software used was R Core Team 2022 (Version 4.2.2).

## RESULTS

The research involved 10 female speech therapists from different regions of Brazil (Center-West, North, Northeast, Southeast, and South). Most of the professionals were between 31 and 40 years old, had postgraduate degrees, and most were specialists. All participants had experience in Orofacial Motricity (OM), evenly distributed across years of experience. The majority worked with infants, and all worked with preschoolers, with varying lengths of experience. Additionally, half of the speech therapists mentioned teaching experience in Speech Therapy (Table 1).

**Table 1:** Characterization of participating speech therapists (personal and professional)

Characteristics (N=10)	Variables	N	%
Gender	Female	10	100
	Male	0	0
Age Group	Between 20 and 30 years	2	20
	Between 31 and 40 years	4	40
	Between 41 and 50 years	2	20
	Over 50 years	2	20
Postgraduate studies	Yes	8	80
	No	2	20
Academic background	Bachelor's degree	2	20
	Specialist	3	30
	Master's degree	2	20
	Doctorate	2	20
	Post-doctorate	1	10
	Associate Professor	0	0
Experience in Orofacial Motricity (MO)	Less than 5 years	2	20
	Between 5 and 10 years	2	20
	Between 11 and 15 years	2	20
	Between 16 and 20 years	2	20
	Between 21 and 25 years	0	0
	Between 26 and 30 years	1	10
	More than 30 years	1	10
Work in the Orofacial Motricity (OM) field (infants)	Does not work	4	40
	Less than 5 years	2	20
	Between 5 and 10 years	0	0
	Between 11 and 15 years	1	10
	Between 16 and 20 years	1	10
	Between 21 and 25 years	1	10
	Between 26 and 30 years	0	0
	More than 30 years	1	10
Work in the Orofacial Motricity (OM) field (preschoolers)	Does not work	0	0
	Less than 5 years	3	30
	Between 5 and 10 years	4	40
	Between 11 and 15 years	0	0
	Between 16 and 20 years	1	10
	Between 21 and 25 years	0	0
	Between 26 and 30 years	1	10
	More than 30 years	1	10
Teaching	Non-teaching	5	50
	Less than 5 years	1	10
	Between 5 and 10 years	2	20
	Between 11 and 15 years	1	10
	Between 16 and 20 years	0	0
	Between 21 and 25 years	0	0
	Between 26 and 30 years	0	0
	More than 30 years	1	10
Region (BR) where the profession is practiced	North	1	10
	Northeast	2	20
	Center-West	1	10
	Southwest	3	30
	South	3	30

**Caption:** N = Number of participating professionals; % = Relative percentage frequency; MO = Orofacial Motricity; BR = Brazil

Most participants reported using instruments in their clinical and research routines in Orofacial Motricity (OM), with the MBGR^(10)^ protocol and the OMES^(11)^ protocol mentioned most frequently (Table 2). During the applicability procedure, professionals used the Orofacial Myofunctional Clinical History with Instruction belonging to the MMBGR Protocol - Infants and Preschoolers, with one to four caregivers, totaling 28 applications. Most speech therapists applied the protocol exclusively in private practice (Table 3).

**Table 2:** List of OM protocols used in clinical practice (assessment or follow-up) and in research routine

**Clinical routine (N)**	**%**	**Research routine (N)**	**%**	**Authors**	**Name**	**Objective**	**Age group**
5	50	3	30	Katia Flores Genaro, et al. (2009)	Avaliação Miofuncional Orofacial: protocolo MBGR^(10)^	Establish assessment, diagnosis, and prognosis in Orofacial Myofunctional Therapy	Children aged 6 to 12 years old.
1	10	1	10	Andréa Monteiro Correia Medeiros, et al. (2022)	Protocolo MMBGR – Lactentes e Pré-escolares^(6)^	Establish assessment, diagnosis, and prognosis in Orofacial Myofunctional Therapy	Children between 6 and 71 months old (Infants and Preschoolers).
1	10	1	10	Cristina Ide Fujinaga, et al. (2013)	Preterm Oral Feeding Readiness Assessment Scale (POFRAS)^(12)^	Assist health professionals in determining the appropriate timing to initiate breastfeeding in premature infants	Corrected gestational age ≤36 weeks and 6 days (Premature infants).
2	20	2	20	Cláudia Maria de Felício, et al. (2008)	Protocolo de Avaliação Miofuncional Orofacial com Escores (AMIOFE)^(11)^	Identify and grade orofacial myofunctional disorders	Children, adolescents, and adults.
1	10	1	10	Participant-authored instrument	Not specified	Not specified	Not specified

**Caption:** N = Number of participating professionals

**Table 3:** Analysis of the number of applications carried out by speech therapists with caregivers of infants/preschoolers, by institution, sector, and time spent on protocol application

	Variables	N	%
Number of applications of the Clinical History Protocol - MMBGR Infants and Preschoolers, with caregivers of infants and preschoolers	1	1	10
	2	1	10
	3	7	70
	4	1	10
	5	0	0
Institution type where the protocol was applied	Public	3	30
	Private	6	60
	Mixed	1	10
Sector of protocol usage	Office / Outpatient Clinic	9	90
	Clinic	1	10
	Hospital	0	0
	Home Care	1	10
	Basic Health Unit	0	0
Time spent to complete the protocol application	Less than 30 minutes	1	10
	Between 30 and 45 minutes	4	40
	More than 45 minutes	5	50

**Caption:** N = Number of participating professionals; % = Relative percentage frequency

The results were analyzed based on the protocol's ease of application and completion, its use in professional practice, and its usefulness for speech therapy clinics. The analysis also considered the usefulness of the instructional part in guiding the protocol completion and the need for prior reading.

The specialists achieved total agreement regarding the ease of application and completion of the Orofacial Myofunctional Clinical History Protocol (Table 4). However, a minority of professionals reported difficulty in understanding the topic “Feeding Development” (Chart 1). Regarding the time, most professionals did not apply the protocol within the estimated duration^(1)^, with half exceeding 45 minutes, and a minority reported applying it in less time (Table 3).

**Table 4:** Percentage of agreement among experts and Content Validity Index regarding the clarity, feasibility, and contribution of the MMBGR Protocol for Infants and Preschoolers - Orofacial Myofunctional Clinical History and Instruction Manual in clinical practice

	Agreements (n)	CVI (%)	p-value
**1. Protocol application ease**	10	100	1,000
**2. Protocol filling ease**	10	100	1,000
**3. Protocol utility in speech therapy clinic**	9	90	0,972
**4. Protocol use in professional practice**	10	100	1,000
**5. Utility of the Instructions for protocol filling**	8	80	0,851
**6. Need for prior reading of the Instructions for protocol application**	7	70	0,617

Exact Binomial Test

**Caption:** CVI = Content Validity Index; N = Absolute frequency; % = Percentage

**Chart 1:** Qualitative record of speech therapists' justifications regarding the applicability of the MMBGR Protocol for Infants and Preschoolers - Orofacial Myofunctional Clinical History and Instruction Manual

Questions and justifications
**The execution of the protocol was easy to apply?**
F6 – There was a question that seemed difficult to understand both for me and for the guardians - in the current feeding item where it asks “most of the time, with whom, how, and where do they eat?”.
**Was the MMBGR Protocol for Infants and Preschoolers - Clinical History easy to fill out?**
F3 - In some sections, I was unsure if it applied to preschoolers or if it was specific to infants, like the “Current feeding pattern” section.
**Did the “Instruction Manual” for the MMBGR Infants and Preschoolers clinical history protocol assist in filling out the protocol?**
F8 - I had some doubts that were not in the instruction manual and left them marked on the forms, especially in section 8.A.
**Do you consider it important for the Instruction Manual of the MMBGR Infants and Preschoolers clinical history protocol to be read before applying the protocol?**
F8 - The clinical history section of the instruction manual doesn't have such specific information that would prevent its use before reading it.
F9 - I found the protocol to be quite self-explanatory.
**Comments inserted in the protocols used with guardians of infants and preschoolers.**
F8 - In the anamnesis/clinical history, we usually include the patient's impressions. Therefore, in this item, I would consider asking the guardian what they think about the general motor development. But I got the impression that in the protocol, I mark whether it's altered or normal based on the expected age. And if the informant doesn't remember, how can I say normal or altered? *The father said: “I don't remember, but everything was normal!” (Motor development);
Do I ask the first items for 5-year-old children too? (Current feeding pattern);
If they never eat using the tablet, do I leave it blank? (Most of the time, with whom, how, and where do they eat - Current feeding).

**Caption:** F6 = Speech Therapist 6; F3 = Speech Therapist 3; F8 = Speech Therapist 8; F9 = Speech Therapist 9; 8.A = Patient A attended by Speech Therapist 8

Regarding the use of the protocol in professional practice, all participants provided a favorable response (Table 4). As for the usefulness for speech therapy clinics, there was a prevalence of agreement among speech therapists, with only one neutral response stating: “neither agree nor disagree” (Table 4).

Most participants agreed on the usefulness of the Instructional part of the MMBGR Protocol - Infants and Preschoolers (Chart 1). Regarding the need for prior reading of the Instructional part for the use of the Orofacial Myofunctional Clinical History Protocol^(2)^, most professionals fully agreed (Table 4). However, some disagreements were noted, mentioning the self-explanatory nature of the protocol and the absence of information preventing the application of the instrument without prior reading (Chart 1).

Other comments on content and appearance were added to the documentation of the applied protocols, such as: inclusion of fields for recording medical record numbers, information about visual acuity, allergies, and daily life routine, school identification, and utensils used for patient feeding supplementation, as well as the profession of caregivers. However, all these considerations encompass aspects already analyzed during the content and appearance validation of the instrument, a stage conducted in a previous study^(2)^.

## DISCUSSION

Given the need to complete all validation steps for a test^(1)^, the importance of conducting the validity based on response processes stage^(7)^ for the MMBGR Protocol - Infants and Preschoolers: Instruction and Orofacial Myofunctional Clinical History^(2)^ was considered. Gathering clinical history in speech therapy practice is highly relevant, especially with validated instruments that serve as good guides for clinical procedures. Therefore, for the validation of the Clinical History protocol and its accompanying Instructional part, aspects of application by speech therapists were considered, considering the characteristics of the target audience.

The participant's research included personal and professional characterization of participating speech therapists, with information indicating a predominance of females in Speech Therapy^(13)^. The participants showed significant experience and a prevalence of postgraduate titles, reflecting their continuous education profile^(14)^, which likely contributed to a more refined and critical analysis of the instrument's application.

The experience in Orofacial Motricity (OM) referred to by all participants may be related to the pursuit of education beyond the generalist nature of academic education in Brazil, followed by a range of specialization possibilities in Speech Therapy^(14)^. The higher number of speech therapists working in OM with preschoolers can be justified by the high demand for speech therapy services in this age group^(15)^. The prevalence of teachers as participants may be related to their active participation in academic research, as well as the interest of speech therapists in teaching^(14)^. The sample of professionals included all regions of the country, indicating the study's reach across different academic and sociocultural backgrounds.

The majority of participating professionals in the study already use protocols in their clinical and research routine (Table 2), indicating familiarity with instrument use and a tendency to better appreciate the utility of the MMBGR Protocol - Infants and Preschoolers. However, this experience can also contribute to a critical and thorough analysis of the protocol. It is worth noting that regardless of using other instruments, 100% of speech therapists stated that they would use the Clinical History protocol in their practice. The total agreement regarding the ease of application and completion of the MMBGR Protocol - Infants and Preschoolers: Instruction and Orofacial Myofunctional Clinical History indicates a favorable opinion on its execution. Despite this, there was a mentioned opinion about the difficulty in understanding the “Feeding Development” topic (Chart 1), with the justification of not specifying the age group for which the items were directed. It is worth mentioning that this topic applies to all age groups and does not require age scaling. Despite this comment by the speech therapist, there was no interference in the validation of the instrument's applicability, given the indices obtained from its final acceptance.

The participant emphasized the importance of protocol usage in professional practice, noting 100% agreement among speech therapists^(2)^ regarding its significance in filling a gap in speech therapy practices, spanning various institutions and service sectors for infants and preschoolers. They also highlighted the protocol's utility for speech therapy clinics, evidenced by a high IVC percentage (90%). The participant considers protocol usability and utility crucial for investigating a subject's history to gather information that guides clinical decision-making^(2)^.

Regarding the average time for applying the clinical history protocol (between 30 and 45 minutes)^(2)^, there was variation in the responses of the participating speech therapists, with one professional applying it more quickly (10%), 4 (40%) within the time specified in the instruction, and/or 5 (50%) taking longer than recommended. This data may be related to the individual approach of each participating speech therapist in clinical practice, including variations in average appointment times and differences in the target audience and application scenarios. It is worth noting that regardless of the application time, all responses indicated the possibility of using the instrument in clinical routine.

The instruction manual, which covers guidance on filling out the Clinical History and Orofacial Myofunctional Examination, corresponding to the MMBGR Protocol - Infants and Preschoolers^(2,6)^, was proposed to serve as a practical guide that directs the understanding and completion of the items presented in the instrument, minimizing potential errors. This is an unprecedented material, still uncommon in the presentation of instruments in speech therapy clinics^(16)^.

The agreement rates are favorable regarding the usefulness of the Instruction Manual (80%) and the need for prior reading (70%) for filling out the instrument, despite the occurrence of discordant responses. It is worth noting that an IVC value of not less than 70% was used as a parameter for agreement. The self-explanatory nature of the clinical history protocol, which was referred to as a disagreement by one speech therapist, contradicts what was already validated in its content in the previous stage of this study^(2)^ (Table 2). Regarding the other non-concordant responses, the comments made by the participants were few, covering content that had already been verified in the content validation stage conducted earlier^(2)^ (Chart 1).

The research presented a limitation related to the delay of participants in adjusting their clinical routine to apply the research instrument with caregivers of infants and/or preschoolers. Justifications for the delay included patient absences and appointment cancellations (especially in private practices), busy schedules with a high demand for appointments (resulting in time constraints for applying the instruments, especially in the public sector), and difficulties in scheduling patients of the required age group as reported by speech therapists. This led to delays in the delivery of requested materials and the completion of electronic forms by participants, even after they had already given a favorable response to participating in the research. It was necessary to extend the initially established deadlines.

## CONCLUSION

The MMBGR Protocol - Infants and Preschoolers: Instruction Manual and Orofacial Myofunctional Clinical History Protocol, part of the MMBGR Protocol - Infants and Preschoolers, designed for the age group of 6 to 71 months old, obtained satisfactory results in the evidence of validity based on response processes, with an IVC value equal to or greater than 70% in all items of the Instruction Manual and between 90 to 100% for the Orofacial Myofunctional Clinical History Protocol.

The Instruction Manual and the Orofacial Myofunctional Clinical History Protocol, belonging to the MMBGR Protocol - Infants and Preschoolers, have demonstrated validity based on response processes for use in speech therapy practice.
