# Supplementary material for: Validity based on the response processes of the MMBGR Protocol Infants and Preschoolers: Instructional and Orofacial Myofuncional Clinical History
Source: Codas. 2024 May 31;36(3):e20230109. doi: 10.1590/2317-1782/20242023109en (PMC11166033; doi:10.1590/2317-1782/20242023109en)
Supplement: Translation

# Translation: Validade baseada nos processos de respostas - Protocolo MMBGR Lactentes e Pré-escolares: Instrutivo e História Clínica Miofuncional Orofacial

**DOI:** 10.1590/2317-1782/20242023109pt

**Authors:** Alexia Stéfanny Menezes da Conceição dos Santos, Yasmim Dourado Goes, Herick Santos Assis, Maria Vanessa Martins Alves, Amanda Tafnes Soares de Melo, Geovania Dias Barbora, Íkaro Daniel de Carvalho Barreto, Elisdete Maria Santos de Jesus, Giédre Berretin-Felix, Andréa Monteiro Correia Medeiros

**Affiliations:** 1 Departamento de Fonoaudiologia, Universidade Federal de Sergipe – UFS - São Cristóvão (SE), Brasil.; 2 Departamento de Fonoaudiologia, Faculdade de Odontologia de Bauru – FOB, Universidade de São Paulo – USP - Bauru (SP), Brasil.; 3 Prefeitura Municipal de São Cristóvão - São Cristóvão (SE), Brasil; 4 Centro Brasileiro de Pesquisa em Avaliação e Seleção e de Promoção de Eventos – CEBRASPE - Brasília (DF), Brasil.; 5 Faculdade de Ciências Farmacêuticas – FCF, Universidade Estadual de Campinas – UNICAMP - Campinas (SP), Brasil.; 6 Programa de Pós-graduação em Ciências da Saúde – PPGCS, Universidade Federal de Sergipe – UFS - São Cristóvão (SE), Brasil.

**Keywords:** Fonoaudiologia, Lactent, Pré-escolar, Estudos de Validação, Terapia Miofuncional, Sistema Estomatognático, Anamnese

## Abstract

**Objetivo:**

Apresentar a etapa da evidência de validade baseada nos processos de respostas do Protocolo MMBGR Lactentes e Pré-escolares: Instrutivo e História Clínica Miofuncional Orofacial.

**Método:**

Estudo desenvolvido conforme recomendações para validação de testes em Fonoaudiologia. Realizada análise da validade baseada nos processos de resposta do instrumento. Participaram dez fonoaudiólogos, que atuam em clínica e/ou pesquisa da Motricidade Orofacial com população entre 6 e 71 meses de idade, que aplicaram o Protocolo MMBGR Lactentes e Pré-escolares: Instrutivo e História Clínica Miofuncional Orofacial junto aos responsáveis pelas crianças. Os fonoaudiólogos emitiram apreciação sobre aplicabilidade do instrumento via formulário eletrônico do Google®, contendo questões dicóticas e/ou múltipla escolha, e escala likert com espaço para justificar respostas negativas. Os dados foram tabulados em planilhas Microsoft Excel 2016® e analisados pelo Índice de Validade de Conteúdo (IVC). Utilizado software R Core Team 2022 (Versão 4.2.2).

**Resultados:**

Todos os itens do Protocolo MMBGR Lactentes e Pré-escolares: Instrutivo e História Clínica Miofuncional Orofacial foram válidos na aplicação em contexto real. Protocolo de História Clínica Miofuncional Orofacial - IVC 100% quanto à facilidade de aplicação e preenchimento, e uso na prática profissional; e IVC 90% quanto à utilidade para clínica fonoaudiológica. O Instrutivo obteve IVC 80% quanto à utilidade e 70% referente à necessidade de leitura prévia para preenchimento do Protocolo MMBGR Lactentes e Pré-escolares.

**Conclusão:**

O Instrutivo e o Protocolo História Clínica Miofuncional Orofacial, pertencentes ao protocolo MMBGR – Lactentes e Pré-escolares tiveram comprovada validade baseada nos processos de resposta, para uso na clínica fonoaudiológica.

## INTRODUÇÃO

O levantamento da história clínica do paciente, por meio da entrevista inicial, é de extrema relevância para a prática clínica fonoaudiológica, a fim de que sejam obtidos dados à respeito da queixa, aspectos do desenvolvimento e fatores biopsicossociais que podem exercer influência sobre o estado de saúde atual do sujeito^(1)^. Essas informações norteiam a conduta, junto ao exame clínico, conforme diagnóstico estabelecido pelo fonoaudiólogo^(1-4)^.

Atualmente, a utilização de instrumentos padronizados na clínica da Motricidade Orofacial (MO) tem possibilitado uma prática quantificável, capaz de promover resultados cada vez mais confiáveis^(5)^. Para a faixa etária entre 6 meses e 5 anos e 11 meses de idade foi proposto o Protocolo MMBGR – Lactentes e Pré-escolares^(2,6)^.

O Protocolo MMBGR – Lactentes e Pré-escolares é composto por Exame Clínico Miofuncional Orofacial (validado quanto ao conteúdo do teste, aplicabilidade e confiabilidade)^(6)^ e pelo Protocolo de História Clínica Miofuncional Orofacial e Instrutivo (validados quanto ao conteúdo e aparência^(2)^, ainda sem realização da etapa de validação baseada nos processos de resposta).

Diante do exposto, o objetivo do presente estudo é apresentar a etapa da evidência de validade baseada nos processos de resposta^(7)^ do Protocolo MMBGR Lactentes e Pré-escolares: Instrutivo e História Clínica Miofuncional Orofacial^(2)^.

## MÉTODO

Estudo descritivo, aprovado pelo Comitê de Ética e Pesquisa em Seres Humanos da Universidade Federal de Sergipe, sob n° CAAE 12529419.6.0000.5546, parecer: 5.249.919. Todos os participantes (profissionais de Fonoaudiologia) e os responsáveis pelos lactentes e/ou pré-escolares assinaram os respectivos Termos de Consentimento Livre e Esclarecido (TCLE).

Trata-se da análise da validade baseada nos processos de resposta do Protocolo MMBGR Lactentes e Pré-escolares: Instrutivo e História Clínica Miofuncional Orofacial^(2)^, conforme diretrizes dos estudos de validação^(7)^. Realizou-se o procedimento de aplicabilidade, a partir do uso do protocolo por fonoaudiólogos (população-alvo)^(7)^, junto aos responsáveis por crianças menores de 6 anos de idade.

Participaram 10 fonoaudiólogos, distribuídos pelas várias regiões do Brasil, os quais foram contatados por meio de aplicativo de mensagens e e-mail, após consulta de seu currículo *lattes,* para verificação sobre formação profissional em Fonoaudiologia e atuação na área de MO. Houve uma sondagem prévia de cada nome conforme visibilidade em redes sociais e/ou serem reconhecidos por atuação na área de MO. Os critérios de inclusão estabelecidos consistiram em: ser fonoaudiólogo(a), com atuação, mesmo que de modo não exclusivo, na área da MO, e trabalhar com população menor de 6 anos de idade. Como critérios de exclusão, considerou-se: ter participado das etapas anteriores do processo de validação do Protocolo MMBGR Lactentes e Pré-escolares; desistir de colaborar com o estudo, atrasar ou não entregar os documentos requeridos.

Ao aceitar participar da pesquisa, cada fonoaudiólogo recebeu o link do formulário eletrônico (Caracterização – parte 1) à respeito da caracterização pessoal e trajetória profissional (gênero, faixa etária, formação acadêmica, experiência em MO, atuação na área de MO com lactentes e/ou pré-escolares, e docência; além da região de atuação no Brasil). Questionou-se, ainda, sobre a utilização de protocolos na rotina clínica e de pesquisa. O Instrutivo e o Protocolo de História Clínica Miofuncional Orofacial^(2)^ pertencentes ao Protocolo MMBGR – Lactentes e Pré-escolares foram disponibilizados por e-mail, em versão pdf.

Todos os fonoaudiólogos participantes foram instruídos a utilizar o instrumento durante a rotina habitual dos atendimentos, sendo em consultórios e/ou clínicas de Fonoaudiologia, setores público e/ou privado. Cada profissional deveria aplicar o instrumento junto a um total de um a quatro responsáveis pelos lactentes e/ou pré-escolares, que consentiram em participar mediante assinatura de TCLE.

Após realização da Anamnese/Entrevista Inicial com o responsável pelo paciente, com o uso do Protocolo MMBGR Lactentes e Pré-escolares: Instrutivo e História Clínica Miofuncional Orofacial, houve a apreciação de cada fonoaudiólogo em formulário eletrônico (Avaliação da aplicação do Protocolo História Clínica MMBGR lactentes e pré-escolares – parte 2), através do link fornecido pelos pesquisadores. Os fonoaudiólogos emitiram seus pareceres conforme previsto na etapa de validação baseada nos processos de resposta^(7)^, considerando, para o protocolo de História Clínica Miofuncional Orofacial: facilidade de aplicação e de preenchimento, uso na prática profissional, utilidade para a clínica fonoaudiológica e o tempo para aplicação. E, para o Instrutivo: utilidade quanto à orientação de preenchimento do protocolo e à necessidade da leitura prévia para aplicação do instrumento.

A partir dos itens avaliados com uso da escala de Likert foram consideradas concordância as respostas correspondentes à: “concordo totalmente” e “concordo parcialmente”, com espaço para justificativa. A alternativa “nem concordo, nem discordo” representou neutralidade quanto ao questionamento. Já as alternativas correspondentes à “discordo parcialmente” e “discordo totalmente” foram consideradas discordâncias.

Os dados coletados via formulários eletrônicos do Google® (partes 1 e 2) foram tabulados em planilhas do Microsoft Excel 2016®. Para análise foi utilizada a estatística descritiva e os resultados apresentados em percentual. As variáveis categóricas descritas por meio de frequência absoluta e relativa percentual. Foi calculado o índice de validade de conteúdo (IVC)^(8)^. Testada a hipótese de não inferioridade do IVC em relação a concordância de 70% por meio do teste exato binomial (Wörz e Bernhardt, 2020)^(9)^. O nível de significância adotado foi de 5% e o software adotado foi o R Core Team 2022 (Versão 4.2.2).

## RESULTADOS

Participaram da pesquisa 10 fonoaudiólogos distribuídos pelas cinco regiões do Brasil (Centro-Oeste, Norte, Nordeste, Sudeste e Sul). Na amostra, todos os profissionais são do sexo feminino, estando a maioria na faixa etária entre 31 e 40 anos. Houve prevalência de profissionais com pós-graduação, e ao serem consideradas as respostas referentes ao maior nível de formação acadêmica obtido pelo profissional, houve predomínio de especialistas. Todos referiram experiência em MO, distribuídos uniformemente em relação ao período em anos. Em relação às faixas etárias, a maioria atua com lactentes, e todos com pré-escolares, com diversos tempos de atuação. Houve ainda metade dos fonoaudiólogos que citaram experiência em docência na Fonoaudiologia (Tabela 1).

**Tabela 1:** Caracterização dos fonoaudiólogos participantes (pessoal e profissional)

Características (N=10)	Variáveis	N	%
**Gênero**	Feminino	10	100
	Masculino	0	0
**Faixa etária**	Entre 20 e 30 anos	2	20
	Entre 31 e 40 anos	4	40
	Entre 41 e 50 anos	2	20
	Mais que 50 anos	2	20
**Pós-graduação**	Sim	8	80
	Não	2	20
**Formação Acadêmica**	Bacharel	2	20
	Especialista	3	30
	Mestre	2	20
	Doutor	2	20
	Pós-Doutorado	1	10
	Livre Docente	0	0
**Experiência em MO**	Menos que 5 anos	2	20
	Entre 5 e 10 anos	2	20
	Entre 11 e 15 anos	2	20
	Entre 16 e 20 anos	2	20
	Entre 21 e 25 anos	0	0
	Entre 26 e 30 anos	1	10
	Mais que 30 anos	1	10
**Atuação na área de MO (lactentes)**	Não atua	4	40
	Menos que 5 anos	2	20
	Entre 5 e 10 anos	0	0
	Entre 11 e 15 anos	1	10
	Entre 16 e 20 anos	1	10
	Entre 21 e 25 anos	1	10
	Entre 26 e 30 anos	0	0
	Mais que 30 anos	1	10
**Atuação na área de MO (pré-escolares)**	Não atua	0	0
	Menos que 5 anos	3	30
	Entre 5 e 10 anos	4	40
	Entre 11 e 15 anos	0	0
	Entre 16 e 20 anos	1	10
	Entre 21 e 25 anos	0	0
	Entre 26 e 30 anos	1	10
	Mais que 30 anos	1	10
**Docência**	Não Docente	5	50
	Menos que 5 anos	1	10
	Entre 5 e 10 anos	2	20
	Entre 11 e 15 anos	1	10
	Entre 16 e 20 anos	0	0
	Entre 21 e 25 anos	0	0
	Entre 26 e 30 anos	0	0
	Mais que 30 anos	1	10
**Região (BR) que exerce a profissão**	Norte	1	10
	Nordeste	2	20
	Centro-Oeste	1	10
	Sudeste	3	30
	Sul	3	30

**Legenda:** N = Número de profissionais participantes; **% =** Frequência relativa percentual; MO = Motricidade Orofacial; BR = Brasil

A maior parte dos participantes, afirmaram utilizar instrumentos na rotina clínica e na pesquisa em MO, sendo o protocolo MBGR^(10)^ e o protocolo AMIOFE^(11)^ citados com maior frequência (Tabela 2). Sobre o procedimento de aplicabilidade propriamente dito, os profissionais utilizaram o Protocolo de História Clínica Miofuncional Orofacial com Instrutivo, pertencentes ao Protocolo MMBGR – Lactentes e Pré-escolares, com os responsáveis de 1 a 4 crianças, atingindo um total de 28 aplicações. A maioria dos fonoaudiólogos-aplicou exclusivamente na rede privada-(Tabela 3).

**Tabela 2:** Relação de protocolos da MO utilizados na rotina clínica (avaliação ou acompanhamento) e na rotina de pesquisa

**Rotina clínica (N)**	**%**	**Rotina de pesquisa (N)**	**%**	**Autores**	**Nome**	**Objetivo**	**Faixa etária**
5	50	3	30	Katia Flores Genaro, et al. (2009)	Avaliação Miofuncional Orofacial: protocolo MBGR^(10)^	Estabelecer avaliação, diagnóstico e prognóstico em Motricidade Orofacial	Crianças de 6 a 12 anos de idade
1	10	1	10	Andréa Monteiro Correia Medeiros, et al. (2022)	Protocolo MMBGR – Lactentes e Pré-escolares^(6)^	Estabelecer avaliação, diagnóstico e prognóstico em Motricidade Orofacial	Crianças entre 6 e 71 meses (Lactentes e Pré-escolares)
1	10	1	10	Cristina Ide Fujinaga, et al. (2013)	Preterm Oral Feeding Readiness Assessment Scale (POFRAS)^(12)^	Auxiliar profissionais de saúde a determinar o momento adequado para início do aleitamento materno em prematuros	Idade gestacional corrigida ≤36 semanas e 6 dias (Prematuros)
2	20	2	20	Cláudia Maria de Felício, et al. (2008)	Protocolo de Avaliação Miofuncional Orofacial com Escores (AMIOFE)^(11)^	Identificar e graduar distúrbios miofuncionais orofaciais	Crianças, jovens e adultos
1	10	1	10	Instrumento de autoria do participante	Não informado	Não informado	Não informado

**Legenda:** N = Quantidade de profissionais participantes

**Tabela 3:** Análise da quantidade de aplicações realizadas por fonoaudiólogo com os responsáveis por lactentes/pré-escolares, da instituição, setor e tempo despendido para aplicação do protocolo

	Variáveis	N	%
**Quantidade de aplicações do Protocolo História Clínica - MMBGR Lactentes e Pré-escolares, com responsáveis por lactentes e pré-escolares**	1	1	10
	2	1	10
	3	7	70
	4	1	10
	5	0	0
**Tipo de instituição que foi realizada a aplicação do protocolo**	Pública	3	30
	Privada	6	60
	Mista	1	10
**Setor de utilização do protocolo**	Consultório/ Ambulatório	9	90
	Clínica	1	10
	Hospital	0	0
	Domiciliar (Home Care)	1	10
	Unidade Básica de Saúde	0	0
**Tempo gasto para realização da aplicação do protocolo**	Menos de 30 minutos	1	10
	Entre 30 e 45 minutos	4	40
	Mais de 45 minutos	5	50

**Legenda:** N = Número de profissionais participantes; **% =** Frequência relativa percentual

Os resultados que foram analisados quanto ao protocolo, seguem descritos conforme facilidade de aplicação e de preenchimento, uso na prática profissional e utilidade para a clínica fonoaudiológica. E, em relação ao instrutivo: a utilidade quanto a orientação de preenchimento do protocolo e a necessidade da leitura prévia.

Quanto a facilidade de aplicação e de preenchimento do Protocolo História Clínica Miofuncional Orofacial, foi obtida total concordância dos especialistas (Tabela 4). No entanto, uma minoria dos profissionais referiu dificuldade na compreensão do tópico “Desenvolvimento da alimentação” (Quadro 1). Sobre o tempo, a maioria não aplicou na duração equivalente à estimada pelo instrumento ^(1)^, sendo que metade dos profissionais excederam 45 minutos e a minoria relatou aplicação em tempo menor (Tabela 3).

**Tabela 4:** Percentagem de concordância entre os especialistas e Índice de Validade de Conteúdo referentes a clareza, viabilidade e contribuição do Protocolo MMBGR lactentes e pré-escolares - História Clínica Miofuncional Orofacial e Instrutivo na prática clínica

	Concordâncias (n)	IVC (%)	valor-p
**1. Facilidade de aplicação do protocolo**	10	100	1,000
**2. Facilidade de preenchimento do protocolo**	10	100	1,000
**3. Utilidade do protocolo na clínica fonoaudiológica**	9	90	0,972
**4. Uso do protocolo na prática profissional**	10	100	1,000
**5. Utilidade do Instrutivo quanto ao preenchimento do protocolo**	8	80	0,851
**6. Necessidade de leitura prévia do Instrutivo para aplicação do protocolo**	7	70	0,617

Teste Binomial Exato

**Legenda:** IVC = Índice de validade de conteúdo; N = Frequência absoluta; % = Percentual

**Quadro 1:** Registro qualitativo das justificativas dos fonoaudiólogos quanto à aplicabilidade do Protocolo MMBGR lactentes e pré-escolares - História Clínica Miofuncional Orofacial e Instrutivo

Perguntas e justificativas
**A execução do protocolo foi de fácil aplicação?**
F6 – Houve uma questão que pareceu de difícil compreensão tanto para mim como para os responsáveis - no item alimentação atual na parte em que questiona “na maioria das vezes, com quem, como e onde faz as refeições”.
**O Protocolo História Clínica - MMBGR Lactentes e Pré-escolares foi de fácil preenchimento?**
F3 - Em alguns blocos fiquei em dúvida se cabia para pré-escolares ou se é um bloco específico de lactentes, como o bloco “Padrão alimentar atual”
**O “Instrutivo” do protocolo de história clínica MMBGR- lactentes e pré-escolares, auxiliou no preenchimento do protocolo?**
F8 - Tive algumas dúvidas que não estavam no instrutivo e deixei assinaladas nos formulários, principalmente no do 8.A.
**Você considera importante que o Instrutivo do protocolo de história clínica MMBGR- lactentes e pré-escolares, seja lido para a aplicação do protocolo?**
F8 - O instrutivo da parte da história clínica não tem informações tão específicas que impeça sua aplicação antes da leitura do mesmo;
F9 - Achei o protocolo bem autoexplicativo.
**Comentários inseridos nos próprios protocolos utilizados com os responsáveis pelos lactentes e pré-escolares.**
F8 - Na anamnese/história clínica, normalmente, colocamos as impressões do paciente. Logo, neste item eu pensaria em perguntar para o responsável o que ele acha do desenvolvimento motor geral. Mas tive a impressão que no protocolo eu marco se está alterado ou normal baseado na idade esperada. E se o informante não lembra, como vou dizer normal ou alterado? *O pai disse: “— Eu não lembro, mas foi tudo normal!” (Desenvolvimento motor);
Pergunto os primeiros itens para crianças de 5 anos também? (Padrão alimentar atual);
Se nunca come utilizando o tablet eu deixo em branco? (Na maioria das vezes, com quem, como e onde faz as refeições - Alimentação atual).

**Legenda:** F6 = Fonoaudiólogo 6; F3 = Fonoaudiólogo 3; F8 = Fonoaudiólogo 8; F9 = Fonoaudiólogo 9; 8.A = Paciente A atendido por fonoaudiólogo 8

Em relação ao uso do protocolo na prática profissional, todos os participantes emitiram resposta favorável (Tabela 4). No que tange à utilidade para a clínica fonoaudiológica, houve prevalência de concordância pelos fonoaudiólogos, com único posicionamento neutro, ao afirmar “nem concordo, nem discordo” (Tabela 4).

Quanto ao Instrutivo do Protocolo MMBGR Lactentes e Pré-escolares, a maioria dos participantes concordou sobre a sua utilidade. Para a resposta discordante, foi apresentada a justificativa de dificuldade em aspecto não abordado no instrutivo (Quadro 1). Quanto à necessidade de leitura prévia do instrutivo para uso do Protocolo de História Clínica Miofuncional Orofacial ^(2)^, a maioria dos profissionais concordaram totalmente (Tabela 4). Entre as discordâncias, foi comentado sobre o caráter autoexplicativo do protocolo e sobre não haver informações que impeçam a aplicação do instrumento sem a realização da leitura prévia (Quadro 1).

Outros comentários sobre conteúdo e aparência foram acrescentados na documentação dos protocolos aplicados, tais como: inserção de campos para registro do número de prontuário, informações sobre acuidade visual, alergias e rotina de vida diária, identificação escolar e utensílio utilizado para complementação alimentar do paciente, além da profissão dos responsáveis. No entanto, todas essas considerações compreendem aspectos já analisados durante a validação de conteúdo e aparência do instrumento, etapa realizada em estudo anterior^(2)^.

## DISCUSSÃO

Dada a necessidade de serem cumpridas todas as etapas de validação de um teste^(1)^, considerou-se a importância de realizar a etapa de validade baseada nos processos de resposta^(7)^ do Protocolo MMBGR – Lactentes e Pré-escolares: Instrutivo e História Clínica Miofuncional Orofacial^(2)^. O levantamento da história clínica na prática fonoaudiológica possui alta relevância, sobretudo com instrumentos validados que se configurem bons norteadores para guiar o proceder clínico. Diante disso, para validação do protocolo de História Clínica e do Instrutivo que o acompanha, foram considerados os aspectos de aplicação pelo fonoaudiólogo, tendo em vista as características do público a que se destina.

Quanto à caracterização pessoal e profissional dos fonoaudiólogos participantes, as informações referentes à totalidade do gênero feminino podem estar atreladas ao número expressivo de mulheres na Fonoaudiologia^(13)^. O tempo de atuação profissional e prevalência de títulos de pós-graduação, evidencia o perfil da formação continuada dos participantes^(14)^, o que pode ter contribuído com a análise ainda mais apurada e crítica sobre a aplicação do instrumento.

A experiência em MO, referida por todos os participantes, pode ter relação na busca de uma formação que vai além do caráter generalista da formação acadêmica no Brasil, seguida da abrangência de possibilidades de especialização na Fonoaudiologia^(14)^. O maior número de fonoaudiólogos atuantes na área de MO com pré-escolares pode ser justificado pela alta demanda de atendimentos fonoaudiológicos com essa faixa etária^(15)^. Já a prevalência de docentes como participantes pode estar relacionada à participação ativa desses profissionais nas pesquisas acadêmicas, bem como o interesse por parte dos fonoaudiólogos em atuar na docência^(14)^. A amostra de profissionais contemplou todas as regiões do país, apontando o alcance do estudo às diferentes formações acadêmicas e socioculturais.

O fato da maioria dos profissionais participantes do estudo já utilizarem protocolos na sua rotina clínica e de pesquisa (Tabela 2) pode delinear familiaridade com o uso de instrumentos, com tendência à melhor apreciação da utilidade do Protocolo MMBGR - Lactentes e Pré-escolares. Por outro lado, essa mesma experiência pode contribuir para uma análise crítica e minuciosa do protocolo. Ressalta-se que, independentemente de usar outros instrumentos, 100% dos fonoaudiólogos afirmaram que usariam o protocolo de História Clínica em sua atuação.

A totalidade de concordância no que se refere à facilidade de aplicação e de preenchimento do Protocolo MMBGR – Lactentes e Pré-escolares: Instrutivo e História Clínica Miofuncional Orofacial explicita o parecer favorável sobre sua execução. Apesar disso, houve uma opinião referida sobre a dificuldade de compreensão para o tópico de “Desenvolvimento da alimentação” (Quadro 1), com a justificativa da não especificação da faixa etária para a qual os itens foram direcionados. Vale dizer que esse tópico diz respeito a todas as faixas etárias, não necessitando de escalonamento por idade. Apesar desse comentário pelo fonoaudiólogo, não houve interferência na validação da aplicabilidade do instrumento, visto os índices obtidos de sua aceitação final.

Quanto ao uso (emprego) na prática profissional, a concordância de 100% reflete a importância e preenchimento de uma lacuna sobre o uso de protocolos para a atuação fonoaudiológica^(2)^, abrangendo diferentes instituições e setores de atendimento, atendendo à faixa etária de lactentes e pré-escolares. Quanto à utilidade (serventia) para a clínica fonoaudiológica, também é evidenciada pela alta percentagem de IVC (90%) obtida. Considera-se a usabilidade e a utilidade dos protocolos como sendo importantes para a investigação da história pregressa do sujeito, para que sejam obtidas informações que irão direcionar a conduta a ser adotada^(2)^.

No que diz respeito ao tempo médio para aplicação do protocolo de história clínica (entre 30 e 45 minutos)^(2)^, houve variação nas respostas dos fonoaudiólogos participantes, com um profissional aplicando mais rapidamente (10%), 4 (40%) no tempo previsto no instrutivo e/ou 5 (50%) utilizando tempo superior ao preconizado. Tal dado pode estar relacionado com a individualidade na condução da atuação clínica de cada fonoaudiólogo participante, incluindo a variação do tempo médio dos atendimentos, bem como às diferenças de público e situações de aplicação. Salienta-se que, independentemente do tempo para aplicação, a totalidade das respostas referiu a possibilidade de uso do instrumento na rotina clínica.

O instrutivo, que abrange orientações quanto ao preenchimento da História Clínica e do Exame Clínico Miofuncional Orofacial, correspondentes ao Protocolo MMBGR – Lactentes e Pré-escolares^(2,6)^, foi proposto para se configurar como um guia prático que norteie a compreensão e o preenchimento dos itens apresentados no instrumento, minimizando possíveis erros. Trata-se de material inédito, sendo ainda pouco frequente na apresentação de instrumentos da clínica fonoaudiológica^(16)^.

Os índices de concordância são favoráveis no que tange à utilidade do Instrutivo (80%) e à necessidade de leitura prévia (70%) para o preenchimento do instrumento, mesmo com ocorrência de respostas discordantes. Lembrando que foi utilizado como parâmetro para concordância o valor de IVC não inferior a 70%. O caráter autoexplicativo do protocolo de história clínica, que foi referido como discordância por um fonoaudiólogo, vai de encontro ao que já foi validado em seu conteúdo, na etapa anterior do presente estudo^(2)^ (Tabela 2). Em relação às demais respostas não concordantes, os comentários realizados pelos participantes foram em pouca quantidade, compreendendo conteúdos que já foram verificados na etapa de validação de conteúdo realizada anteriormente^(2)^ (Quadro 1),

A pesquisa apresentou como limitação a demora dos participantes em ajustar a rotina clínica para aplicar o instrumento de pesquisa junto aos responsáveis pelos lactentes e/ou pré-escolares. Foram apresentadas as seguintes justificativas sobre a demora: faltas e desmarcações de consultas por parte dos pacientes (sobretudo nos consultórios privados), e rotina atribulada com alta demanda de atendimentos (com prejuízo de tempo para aplicação dos instrumentos, sobretudo àqueles que atuam no setor público). Também foram relatadas pelos fonoaudiólogos, dificuldades de agendamento de pacientes da faixa etária necessária. Isso causou morosidade quanto à entrega dos materiais solicitados e ao preenchimento dos formulários eletrônicos pelos participantes, mesmo quando já havia sido emitida a resposta favorável à participação na pesquisa. Foi necessário ampliar os prazos estabelecidos inicialmente.

## CONCLUSÃO

O protocolo MMBGR – Lactentes e Pré-escolares: Instrutivo e História Clínica Miofuncional Orofacial, pertencente ao protocolo MMBGR – Lactentes e Pré-escolares, destinado a faixa etária de 06 a 71 meses de vida, obteve resultados satisfatórios na evidência de validade baseada nos processos de resposta, tendo obtido valor de IVC igual ou superior a 70% em todos os itens do Instrutivo e entre 90 a 100% quanto ao Protocolo de História Clínica Miofuncional Orofacial.

O Instrutivo e o Protocolo História Clínica Miofuncional Orofacial, pertencentes ao protocolo MMBGR – Lactentes e Pré-escolares tiveram comprovada validade baseada nos processos de resposta, para uso na prática fonoaudiológica.
